# A preference-based item response theory model to measure health: concept and mathematics of the multi-attribute preference response model

**DOI:** 10.1186/s12874-018-0516-8

**Published:** 2018-06-22

**Authors:** Catharina G. M. Groothuis-Oudshoorn, Edwin R. van den Heuvel, Paul F. M. Krabbe

**Affiliations:** 1 Department of Health Technology and Services Research, Faculty of Behavioural, Management and Social Sciences, Technical Medical Centre, University of Twente, PO Box 217, 7500 AE Enschede, The Netherlands; 2 Department of Mathematics and Computer Science, Eindhoven University of Technology, PO Box 513, 5600 MB Eindhoven, The Netherlands; 3 University Medical Center Groningen, Department of Epidemiology, University of Groningen, PO Box 30.001, 9700 RB Groningen, The Netherlands

**Keywords:** Health-related quality of life, Health status, Latent logistic test model, Patient-reported measurement, Rasch model

## Abstract

**Background:**

A new patient-reported health measurement model has been developed to quantify descriptions of health states. Known as the multi-attribute preference response (MAPR) model, it is based on item response theory. The response task in the MAPR is for a patient to judge whether hypothetical health-state descriptions are better or worse than his/her own health status.

**Methods:**

In its most simple form MAPR is a Rasch model where for each respondent on the same unidimensional health scale values are estimated of their own health status and values of the hypothetical comparator health states. These values reflect the quality or severity of the health states. Alternatively, the respondents are offered health-state descriptions that are based on a classification system (e.g., multi-attribute) with a fixed number of health attributes, each with a limited number of levels. In the latter variant, the weights of the levels of the attributes in the descriptive system, which represents the range of the health states, are estimated. The results of a small empirical study are presented to illustrate the procedures of the MAPR model and possible extensions of the model are discussed.

**Results:**

The small study that we conducted to illustrate the procedure and results of our proposed method to measure the quality of health states and patients’ own health status showed confirming results.

**Conclusions:**

This paper introduces the typical MAPR model and shows how it extends the basic Rasch model with a regression function for the attributes of the health-state classification system.

**Electronic supplementary material:**

The online version of this article (10.1186/s12874-018-0516-8) contains supplementary material, which is available to authorized users.

## Background

Health is a sociocultural construct encompassing a wide range of phenomena, so it is not surprising that various actors define it differently. Traditionally, physicians have been guided by a biomedical model and have thus understood health predominantly as a condition that falls within acceptable biological norms. Nowadays, there is an increased awareness of the impact of health and health care on the quality of human life. The conventional clinical health-status construct is now often extended to psychological and even social factors, thereby making subjective measures such as (perceived) health status or ‘quality of life’ necessary — and rightly so, because the ultimate goal of all health interventions is to improve a patient’s perceived health condition. The use of these subjective measures has proliferated ever since the World Health Organization published its definition of health in 1946 [1].

There are several ways to express health. We can compile a ‘snapshot’ of a patient’s current health condition from an ‘image bank’ comprised of health states. These health states consist of discrete health attributes (e.g., domains, dimensions, items) each with a number of levels. When combined, they represent a description of a person’s health status or health-related quality of life (HRQoL) [2]. Subsequently, such health-state descriptions can be measured (valued) by assigning meaningful numbers (values) to an individual’s health state. ‘Meaningful’ is here defined as values that reflect the patients’ health status in relationship to other health states. This is different from subjective measures (e.g., visual analogue scale) that reflect the perception of how individuals experience their health status in relationship to their own internal standards. It is convenient to express individuals’ health in single metric values, as these can be used in health outcomes research, for clinical monitoring of the health status of patient groups, and in particular, in disease modeling studies and economic evaluations.

To obtain health-state values (variously called preferences, utilities, index, or weights), the health-state descriptions must be quantified in terms of seriousness or quality. Differences between health states values are assumed to correspond to increments of quality differences between these states, which implies that the values are on an interval-level scale [2]. Most conventional methods of measurement (or valuation) stem from health economics (e.g., standard gamble, time trade-off) and are susceptible to many disturbing factors such as adaptation, time preference, context, reference point, and other biases [3–5]. To control for adaptation, which occurs in most of these conventional methods (especially for chronically ill patients), all economic valuation methods use hypothetical health states that are assessed by a sample of (unaffected) members of the general population. However, it is reasonable to assume that healthy people are not adequately informed or lack the imagination to appropriately judge the impact of health states, particularly severe ones [6, 7].

A new way to quantify health states was recently introduced. This measurement method, the multi-attribute preference response (MAPR) model, is based on the Rasch model (an item response theory model) [8]. The MAPR model more or less mimics the situation of a patient with a certain health condition lying in a wardroom where the other occupants have (related) complaints and symptoms. This patient is asked to compare his own health state to that of his roommates by indicating whether his own state is better or worse. The conventional preference-based measurement methods usually yield an opinion on health states from healthy controls, while the result of the MAPR is an internal positioning of a patient’s health status with respect to other health states. The response mechanism of the MAPR model is less susceptible to various biases that conventional methods are prone to. Moreover, the MAPR is the first generic health preference model that is fully based on patient perception and reporting; as such it is a genuine patient-reported outcome measure. Apart from being grounded in a renowned measurement theory, the MAPR response tasks are attractive and easy to perform in a self-completion setting.

This article introduces and explains the MAPR model conceptually and mathematically. The first section looks into the background of its measurement mechanism, namely the Rasch model, and expands on its operation in a health setting. The second section describes the MAPR model; the third works through its estimation procedures. Finally, the results of a small empirical study are presented to illustrate the procedures of the MAPR model and possible extensions of the model are discussed.

## Methods

### Measurement mechanism

A probabilistic measurement model was invented by the Danish mathematician Georg Rasch. While primarily employed to assess educational attainment, it is increasingly used for other purposes [9]. Its original setting was the field of reading skills, where it was intended for use with dichotomous response data (e.g., correct/wrong). Nowadays, the Rasch model or the closely related one-parameter logistic model (OPLM) is considered a variant of the class of item response theory (IRT) models [9, 10]. The Rasch model is built around the idea that the probability of a correct response to an item is modeled as a logistic function of the difference between the difficulty of an item (parameterized by *β*) and the characteristics of a person (e.g., ability parameter *θ*):

$$ \pi =P\left(+|\beta, \theta\ \right)=\frac{1}{1+{e}^{\beta -\theta }}=\frac{e^{\theta -\beta }}{1+{e}^{\theta -\beta }}. $$

The Rasch model poses three stringent requirements. The first is *unidimensionality*: a unique one-dimensional latent variable explains the response to the items. The second is *monotonicity*: the probability of a positive response to an item is a non-decreasing function of the latent variable. And the third is *local independence*: for any given individual, the item responses are independent conditional on where individuals are on the underlying latent scale.

**Fig. 1:**
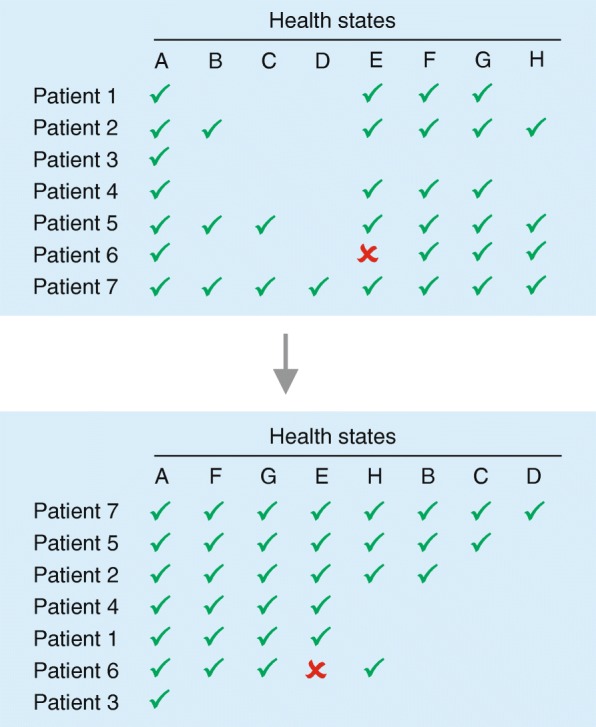
Schematic illustration of the Guttman/Rasch data structure. Representation of the raw data (top) and after sorting of the columns (health states) and the rows (patients) in order to arrive at the hierarchical Guttman/Rasch data structure (a check indicates that this health state is preferred over the next health state, a cross indicates a misfit)

Under the Rasch model, a Guttman scale is the most likely response pattern for a person when items are ordered from least difficult to most difficult [11]. This means that if someone responds correctly to an item, then that person should succeed on all easier items; conversely, if one responds incorrectly, then he/she should fail on all items that are more difficult (Fig. 1). Unlike the Guttman scale, the Rasch model is a probabilistic model. In the latter, the probability that any person will succeed on an easier item will always be greater than the probability of success on a more difficult item. The Guttman scale is the deterministic limiting case of the Rasch model.

### Health context

In the context of health measurement assuming the Rasch model implies that the more positive the difference between the value (the perceived quality) of the health status of a patient (*θ*) and the value of another health state (*β*) to be judged, the higher the probability that the patient will indicate that his/her current health status is better than the presented health state. Or the other way around, patients in very poor health will consider many other health states as better than their own. Using the Rasch model, one can estimate the health status of individual patients (i.e., their ability, in Rasch terminology) and the value of the hypothetical health states (i.e., difficulty of the parameters of items) on the same latent scale. In short, patients are asked to respond to hypothetical health states by comparing these with their own health status. For example, “Is this health state better than your own health state?”

**Fig. 2:**
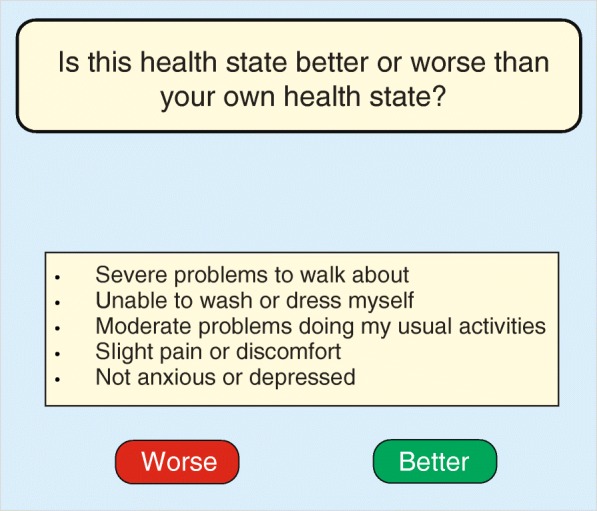
Example of a response task under the multi-attribute preference response (MAPR) model for a multi-attribute health-state description (state ‘33221’) based on the EQ-5D-3 L instrument (3-level version)

In the Rasch model, patients compare their own health status with a few prescribed hypothetical health states. These comparator health states can span the whole continuum from bad to mild (as done in this article in our small empirical study), but they can also denote health states that are closely positioned on the latent scale to the actual health status of the individual patient. Such comparator health states may be based on holistic descriptions or objects. Holistic refers to unstructured verbal descriptions or objects such as people’s faces or skin in photos. In general, holistic objects are often extremely easy to compare and judge. However, features (attributes) by which to describe the object specifically are often absent. Alternatively, health descriptions may be derived from a classification system with multiple attributes, whereby each attribute has a limited number of levels (Fig. 2). The latter approach enables the investigator to predict values for health states that are not part of the empirical study (see below).

Let *θ*_*p*_ be the (unknown) health status of person *p (p = 1, ..., P)*. Suppose that *β*_*i*_ is the (unknown) value of health state *i* (*i = 1, …, I*) as measured on a latent scale. Imposing the logistic function of the difference between a person’s health status and the values of the comparator health states on the probability that a person prefers his/her own health state over the comparator description leads to the Rasch model. More formally, let *Y*_*ip*_ be a random variable with a value of one if a person prefers his/her health status over the hypothetical health state and zero otherwise. In this way it is assumed that the health status *θ*_*p*_ of person *p* is on the same latent scale as the health states *i* with *β*_*i*_ and that a person will most likely prefer his/her own health status over health state *i* if *θ*_*p*_ exceeds *β*_*i*_*.* Under the Rasch model we assume that

1 $$ {\pi}_{ip}=P\left({Y}_{ip}=1\right|{\theta}_p,{\beta}_i\Big)=\frac{e^{\theta_p-{\beta}_i}}{1+{e}^{\theta_p-{\beta}_i}} = \frac{1}{1+{e}^{\beta_i-{\theta}_p}}, $$

or equivalently in the logit form

2 $$ {\eta}_{ip}={\theta}_p-{\beta}_i, $$

where *η* = log (*π*/(1 − *π*)) is the logit link function. This means that if a person’s health status *θ*_*p*_ is equal in quality to the hypothetical comparator health state, so there is no preference difference for either state, the probability of choosing the one over the other is fifty-fifty. Also, the further apart the person’s health state is from the comparator, the larger the probability that the better state is preferred and chosen. In the following, model (1) will be called the holistic MAPR model.

The holistic MAPR model, like the original Rasch, is a descriptive model. It describes the individual patient’s health state (e.g., by localizing patients on the health scale) and the value of the judged comparator states without explaining either of these by characteristics of the patients or the health states. The holistic MAPR is both feasible and attractive in many clinical situations where characteristics cannot be easily discerned, such as body and skin deformations. Comparing and assessing pictures or movies may then be more appropriate. A crucial requirement is that the respondents should be located along the whole health scale from very severe to almost perfect health; otherwise, the model cannot be sufficiently estimated. The typical response task of the MAPR model precludes responses from a sample of the general population. The latter are predominantly healthy and therefore do not provide the information needed to estimate the model.

### MAPR model

Several simple classification systems have been developed to capture the major features of health such that they can be used to describe health states. Each system transposes those features into a certain number of health attributes. The health state can then be measured with a discrete response scale for each attribute at a certain number of levels. For example, the classification system of the preference-based EQ-5D-3 L instrument consists of five health attributes: mobility, self-care, usual activities, pain/discomfort, and anxiety/depression, with a value of 1 (best), 2, or 3 (worst) for each attribute [12]. In this way an EQ-5D-3 L health state can be represented by five digits, with 11111 denoting perfect health and 33333 the worst possible condition. The three-level version of the EQ-5D-3 L system defines 3^5^ = 243 possible partially ordered different health states. The SF-6D health-state classification contains six attributes, namely physical functioning, role limitation, social functioning, pain, mental health, and vitality. With response categories ranging from four to six levels, the SF-6D can describe 18,000 different health states. Some other classification systems are the Health Utilities Index version 3 (HUI-3), 15D, Assessment of Quality of Life (AQoL), and the Quality of Well-Being scale (QWB) [13–16].

### Formal representation

Assume that we now have a classification system wherein a health state is represented as a vector ***x***_*i*_ = (*x*_*i*1_, ..., *x*_*iJ*_) with discrete levels on each of the *J* attributes. The number of levels in the *j*th attribute is denoted by *n*_*j*_, so on attribute *j* the possible values are *1,2, …, n*_*j*_. In this way the vector *(1,1,…,1)* represents perfect health and *(n*_*1*_*, n*_*2*_*, …, n*_*J*_*)* the worst state. Suppose that the value *β*_*i*_ of health state ***x***_***i***_ can be described as a function *β*_*ι*_ = *f (****x***_*i*_*)* to reflect the partial ordering of the health states. In the literature several functions have been proposed to model the value of health states as a function of a set of health attributes. For instance, the simple additive linear model assumes that linearity is present in each attribute and that the value drops by the same amount, for example when moving from level 1 to 2 or from level 2 to 3. A less restrictive and more realistic model can be obtained by taking each attribute as a categorical variable in the regression model:

3 $$ {\beta}_i=f\left({\boldsymbol{x}}_{\boldsymbol{i}}\right)=\sum \limits_{j=1}^J\sum \limits_{k=1}^{n_j}{\alpha}_{jk}{d}_{jk}\left({x}_{ij}\right), $$

where *d*_*jk*_*(x*_*ij*_*)* is a dummy variable with *d*_*jk*_*(x*_*ij*_*) = 1* if *x*_*ij*_ *= k* and zero otherwise. The contribution to the value *β*_*i*_ of health state *x*_*i*_ of a change in attribute *j* from level 1 to *k* is parameterized by *α*_*jk*_. Notice that the regression equation in (3) has no intercept as this parameter is redundant. Furthermore, additional restrictions on *α*_*jks*_ are required for enforcing the partial ordering on the *β* s and for identifying the parameters.^1^ Substituting linear expression (3) for *β*_*i*_ in the logistic expression (2) gives

4 $$ {\eta}_{ip}={\theta}_p-\sum \limits_{j=1}^J\sum \limits_{k=1}^{n_j}{\alpha}_{jk}{d}_{jk}\left({x}_{ij}\right).\kern5.25em $$

The parameterization of the value of health states is not limited to the main effects of the health attributes, as interactions between health attributes can be incorporated in (4) by adding products of (dummies of) health attributes. For identification purposes, the number of parameters should be less than the number of health states that the respondents are asked to compare. In the IRT literature this type of (item/health explanatory) model is called the linear logistic test model (LLTM). It was originally proposed by Scheiblechner [17] and later formalized by Fisher [10, 18–20]. LLTM differs from the Rasch model in that the influence of the quality/severity of the comparator health states is reduced to a linear combination of a fixed number of health-state attributes or interactions between those attributes, with fewer parameters than hypothetical health states. The effects of the attributes and their levels on the health states are estimated instead of the holistic health-state parameters themselves (Formula 1). Being more restrictive than the Rasch model, it enables one to predict values for the complete set of health states that can be constructed for a specific classification system, so predictions can also be made for health states that are not evaluated in the response study.

Suppose we have a sample of *n* patients who compared the same *m* health states *β*_*i*_*(i = 1,...,m)* with their own health status. By substituting the parameterization of the items in terms of their attributes as formulated in (3) into formula (1), the holistic MAPR model, we can write the probability of response of patient *p* on health state *i* as:

5 $$ {\pi}_{ip}=P\left({Y}_{ip}=1\right|{\theta}_p,\beta =f\left({x}_i\right)\Big)=\frac{1}{1+{e}^{-{\theta}_p+\sum \limits_{j=1}^J{\sum}_{k=1}^{n_J}{\alpha}_{jk}{d}_{jk}\left({x}_i\right)}\ }\kern0.5em $$

Model (5) will be denoted as the MAPR model. Estimation of the health state parameters of the MAPR model now boils down to estimation of the parameters *α*_*jk*_*.* In that way, the value of a health state is reflected in the characteristics of the health states as parameterized with the variables *d*_*jk*_(*x*_*i*_).

### Adaptive MAPR model

**Fig. 3:**
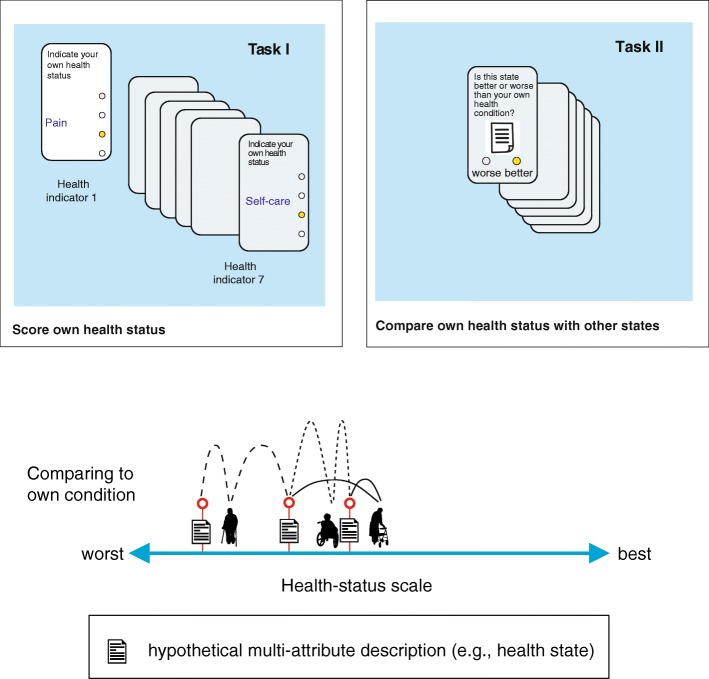
MAPR measurement mechanism

A more adaptive approach is possible. Patients are thereby asked to complete a multi-attribute classification (e.g., EQ-5D-3 L) in advance to classify their own health status, denoted ${\overset{\sim }{\boldsymbol{x}}}_p=\left({\overset{\sim }{x}}_{p1},..,{\overset{\sim }{x}}_{pJ}\right)$. Then, to perform the MAPR response task, they are confronted with a set of (individualized) hypothetical (comparator) health states that were selected in light of the classification of the patients’ own health state from the first task (Fig. 3). So, in this case patients are shown different subsets of health states, depending on${\overset{\sim }{\boldsymbol{x}}}_p$. In principle, this approach allows more precise estimation of the position of the patients’ health status. It also precludes selecting a restricted set of predetermined comparator states to be judged. However, it complicates the analysis of the data, as the subset of presented health states differs between the respondents and depends on the person’s own health state, which is restricted to ${\theta}_p=f\left({\overset{\sim }{\boldsymbol{x}}}_p\right)$. This adaptive operation of the MAPR model is almost similar to computerized adaptive testing (CAT) that is used for standard IRT models. The difference is that for standard IRT models a routine on a central server determines, from a large item bank of candidate items, the next item offered to an individual respondent. For the MAPR model a simple routine as part of a mobile application (www.healthsnapp.info) determines the comparator states (comprising multiple attributes/items) to be assessed by individual patients.

### Estimation of the Rasch model

When assessing health states holistically (i.e., no parameters for the levels of the attributes) as in traditional item response theory, it is assumed that the responses to health states are independent of one another, which gives rise to the following likelihood:

6 $$ L\left(\theta, \beta |{Y}_{ip}={y}_{ip}\right)=\prod \limits_{p=1}^P\prod \limits_{i=1}^I{\pi_{ip}}^{y_{ip}}{\left(1-{\pi}_{ip}\right)}^{1-{y}_{ip}} $$

The parameters of the standard Rasch model can easily be estimated by several methods, e.g. full maximum likelihood estimation, conditional maximum likelihood and marginal maximum likelihood. All of these are based on maximum likelihood estimation or Bayesian estimation, and several procedures have been described in the literature [21].We will describe now the conditional maximum likelihood (CML) estimation.

Let ${R}_p=\sum \limits_{i=1}^I{Y}_{ip}$ be the number of health states that a patient *p* has compared to his own and were considered worse. This number is a sufficient statistic for estimating the patient’s own health state *θ*_*p*_. Thus, the conditional likelihood of the responses is independent of *θ*_*p*_ if we condition on *R*_*p*_*.* This leads to the (CML) estimation equations, after maximizing the likelihood:

7 $$ \sum \limits_{p=1}^P{Y}_{ip}=\sum \limits_{p=1}^PP\left({Y}_{ip}=1\right|{R}_p={r}_p,{\beta}_i\Big)\kern1em \mathrm{for}\ \mathrm{i}=1,..,\mathrm{I}. $$

P (*Y*_*ip*_ *=* 1*|R*_*p*_ *= r*_*p*_*, β*_*i*_) is the probability that the patient’s health status is better than health state *i*, given the number of health states found to be worse than the patient’s health state. These *I-1* equations can be solved using a Newton-Raphson procedure leading to consistent point estimates for the health-state parameters *β*_*i*_.

An estimate of the patient’s own health state *θ*_*p*_ can be obtained with a maximum likelihood estimation procedure. In this second step, the conditional maximum likelihood estimates of *β*_*i*_ are assumed to be fixed and are substituted in the estimation Eq. (6). In this way the uncertainty associated to these estimates is not accounted for. One way to incorporate this uncertainty could be to use a Bayesian estimation method. In that case a sample from the posterior distributions of the item person parameters can be used instead of imputing only the estimates itself [22].

The variance of the ML estimates equals:

$$ Var\left({\widehat{\theta}}_p\right)=\frac{1}{I\left({\widehat{\theta}}_p\right)}=\frac{1}{\sum_{i=1}^I{P}_i\left({\widehat{\theta}}_p\right)\left(1-{P}_i\left({\widehat{\theta}}_p\right)\right)} $$

Note that the estimated *βs* are not incorporated in this variance*.* The maximum of the function *f (x) = x (1-x)* is 0.25 for *x = 0.5.* One can thus see that individual health status can be estimated more precisely when patients have to compare health states that are close to their own state.

For the one parameter logistic model (OPLM), the parameter estimates obtained using CML and marginal maximum likelihood (MML) are usually close. The advantage of CML over the MML procedure is that no a priori assumptions have to be made about a person’s health-state distribution. When this a priori distribution is misspecified, the MML estimates may be biased. It is expected that the distribution of person’s health states is not normally distributed but typically skewed to the right [23]. On the other hand, it has to be underlined that CML estimation also has some pitfalls, such as the fact that individuals with perfect or zero scores do not provide any information and, missing observations can lead to biases in case of missing not completely at random.

Whether a Rasch model fits the data, thereby yielding a unidimensional scale, can be tested with Andersen’s likelihood ratio test [24]. Note that obeying a Rasch model is a sufficient but not a necessary condition of unidimensionality.

### Estimation of the MAPR model

Estimation of the LLTM model is similar to estimation of the Rasch model. Both procedures are based on the fact that the number of worse health states per person is a minimal sufficient statistic for *θ*_*p*_. As a consequence, the parameters *α*_*jk*_ can be estimated without knowledge of the patient’s health status (known as person-free item assessment). Instead, finding the values for β_*I,*_ that maximize the (conditional) likelihood estimation of the LLTM model, boils down to finding the values for *α*_*jk*_.

Both the existence and uniqueness of the CML estimates depend on whether the data matrix is well conditioned. A response matrix is said to be well conditioned if in every possible partition of the health states into two non-empty subsets some patients have given a response of one on some health state in the first set and a response of zero on some health state in the second set [25, 26].

The fit of the MAPR model (LLTM) can be compared with the fit of the Rasch model by using a likelihood ratio test. The deviance of − 2 log-likelihood of the two nested models is approximately Χ^2^- distributed with df = difference between the number of parameters in the two models [18, 27]. When this test is significant, there is evidence that health states are not sufficiently described by the characteristics of the health states as parameterized with the variables *d*_*jk*_(*x*_*i*_). In case there is no statistically significant difference between the Rasch model and the MAPR model, the latter can be used to describe the values of health states. Different formulations of LLTM models can also be compared by performing a likelihood test, as long as these models are nested.

### Empirical study

#### Respondents

The aim of the empirical study was to show first exploratory results in testing the MAPR model. In order to do so we used data from a previously published study that aimed to explore discrepancies in values for health states between the general population and patients that experience specific illness [7]. For this study we used only the data of the patients (*n* = 75).

Two patient groups from the Radboud University Nijmegen Medical Center (Netherlands) participated in that study, which was approved by the Central Committee on Research Involving Human Subjects (region Arnhem-Nijmegen) [7]. One group included patients who were diagnosed with cancer within a time frame of 4–6 weeks before they participated in the study. The other group consisted of chronically ill patients living with the symptoms of rheumatoid arthritis (RA) for at least 3 years. The study protocol was administered face-to-face by a trained interviewer at the homes of the patients.

This initial sample was extended by including patients with a cerebrovascular accident (CVA) or inflammatory bowel disease (IBD) from the hospital Medisch Spectrum Twente (*n* = 35) and patients with liver disease or paraplegia from the University Medical Center Groningen (*n* = 53). The Medical Ethics Review Committee Twente (METC/14124) and Medical Ethics Review Committee UMCG (METC 2015/496) declared that this latter part of the research did not fall under the Medical Research Involving Human Subjects Act.

#### Study design

In the initial study (Radboud) the judgmental task consisted of ranking 17 EQ-5D-3 L health states, supplemented with the patient’s own EQ-5D-3 L description, ‘dead’, and state ‘11111’. Each patient ranked the same 20 health states by putting the card with the ‘best’ health state on top and the ‘worst’ at the bottom. Additionally, the patients unknowingly assessed their own health status in the judgmental task, as their own EQ-5D-3 L health-state description had been incorporated in the set, but they did not assess the health states of the other participants. The task in the other two studies was slightly different (patients did not assess their own health status), but is not likely affecting the results in the empirical study as described in this article. Respondents in the latter two studies were asked to compare the same 17 EQ-5D-3 L health states from the Radboud study with their own health (not explicitly represented in terms of the EQ-5D-3 L description) and express if the EQ-5D-3 L health states was worse or better than their own health status. In all three studies, the EQ-5D-3 L health states were presented in random order to control for potential biases due to presentation order or respondent fatigue.

#### Analysis of the empirical study

First, we fit the Rasch model to the ranking data. Next, we analyze the following (MAPR) model for the value of health state *β*_*i*_*:*

8 $$ {\beta}_i=\sum \limits_{j=1}^5\sum \limits_{k=1}^3{\alpha}_{jk}{d}_{jk}\left({x}_{ij}\right), $$

a model with only main effects for all attributes (with dummy variables). To ensure identification of the parameters *α*_*jk*_, an additional restriction has to be put on these parameters; in this case we choose *α*_*jk*_ = 0 for  *k* = 1, *j* = 1, . . , 5.

Goodness of fit for the holistic MAPR (i.e., the Rasch) model is tested with the Andersen LR test [28]. Then, MAPR model (8) and the Rasch (i.e., the holistic MAPR) model are compared (LR test, correlation coefficient). Next, for every patient the predicted value of its health state following from the estimated MAPR model (8) are calculated based on the patient’s own EQ-5D-3 L description. For every health state shown to the patient, it is determined whether the patient’s estimated health-status value outperforms (i.e., is preferred by the patient) the estimated value of the shown comparator health state. These predicted preferences will then be compared with the observed preferences using a kappa coefficient as measure of agreement. A kappa larger than 0.75 is considered excellent and between 0.4 and 0.75 fair to good [29]. The eRM package in R was used to estimate the MAPR models (LLTM model) and the Rasch model [30].

## Results

In total 163 patients were interviewed for this study. Of these, 48 were cancer patients (34 colorectal cancer, 14 breast cancer) and 42 had a liver-related disease or transplant. The number of participating RA patients was 27. The mean age differs across the participating hospitals, with the oldest patients coming from the Radboud Medical Center (Table 1). Overall, some or major problems were reported for pain (60.1%) and the least problems were reported for self-care (17.8%). Major or severe problems for self-care and mood were reported only by patients with liver-related disease or transplant or by paraplegic patients (Table 2). As the distribution of the health states across the study sites shows, the UMCG had more patients with a severe health condition. But there was a reasonable spread over the whole HRQoL continuum for the three hospitals (Additional file 1).

**Table 1:** Characteristics and evaluation assessment of the study population (*n* = 163)

	Radboud^a^(*n* = 75)	MST (*n* = 35)	UMCG (*n* = 53)
Mean Age, yrs. (sd)	63.6 (9.4)	53.0 (21.4)	48.3 (17.8)
Gender (%)			
Female	36 (50.0)	20 (57.1)	27(50.9)
Male	36 (50.0)	15 (42.9)	26 (49.1)
Diagnosis (%)			
Liver transplant			15 (28.3)
Liver-related disease?			27 (50.9)
CVA		13 (37.1)	
IBD		22 (62.9)	
Cancer	48 (64.0)		
RA	27 (36.0)		
Paraplegic			9 (17.0)
Other/Unknown			2 (3.8)
Education (%)			
Lower	41 (54.7)	6 (17.1)	19 (35.8)
Middle	15 (20.0)	19 (54.3)	6 (11.3)
Upper	19 (25.3)	10 (28.6)	20 (37.7)
Other			8 (15.1)
Mean EQ VAS (sd)	75.2 (14.7)	68.5 (13.5)	72.1 (17.5)
Difficulty assessment (%)			
Very easy	–	10 (28.6)	9 (17.0)
Easy	–	16 (45.7)	17 (32.1)
Neutral	–	5 (14.3)	20 (37.7)
Difficult	–	2 (5.7)	6 (11.3)
Very difficult	–	2 (5.7)	1 (1.9)

^a^Radboud = Radboud University Nijmegen Medical Center,

*MST* hospital Medisch Spectrum Twente, *UMCG* University Medical Center Groningen

**Table 2:** Marginal distribution of patients’ own classification of their health status based on the five attributes, each with three levels, of the EQ-5D-3 L instrument (*n* = 163)

EQ-5D-3 L attributes and levels	Radboud^a^(*n* = 75)	MST (*n* = 35)	UMCG (*n* = 53)
Mobility			
No problems (1)	45 (60.0)	20 (57.1)	29 (54.7)
Some problems (2)	30 (40.0)	15 (42.9)	18 (34.0)
Confined to bed (3)			6 (11.3)
Self-care			
No problems (1)	63 (84.0)	31 (88.6)	40 (75.5)
Some problems (2)	12 (16.0)	4 (11.4)	10 (18.9)
Unable to (3)			3 (5.7)
Usual activities			
No problems (1)	38 (50.7)	12 (34.3)	26 (49.1)
Some problems (2)	34 (45.3)	21 (60.0)	24 (45.3)
Unable to (3)	3 (4.0)	2 (5.7)	3 (5.7)
Pain/Discomfort			
No (1)	34 (45.3)	13 (37.1)	18 (34.0)
Moderate (2)	36 (48.0)	21 (60.0)	32 (60.4)
Extreme (3)	5 (6.7)	1 (2.9)	3 (5.7)
Depression/Anxiety			
Not (1)	59 (78.7)	25 (71.4)	36 (67.9)
Moderately (2)	16 (21.3)	10 (28.6)	12 (22.6)
Extremely (3)			5 (9.4)

^a^*Radboud* Radboud University Nijmegen Medical Center, *MST* hospital Medisch Spectrum Twente, *UMCG* University Medical Center Groningen

**Fig. 4:**
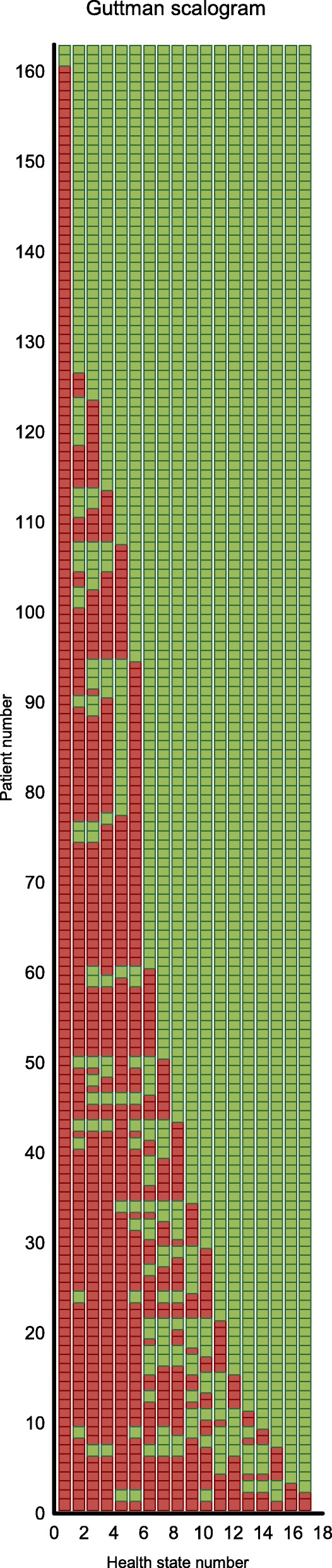
Guttman scalogram (green dots between red ones show misfit)

**Fig. 5:**
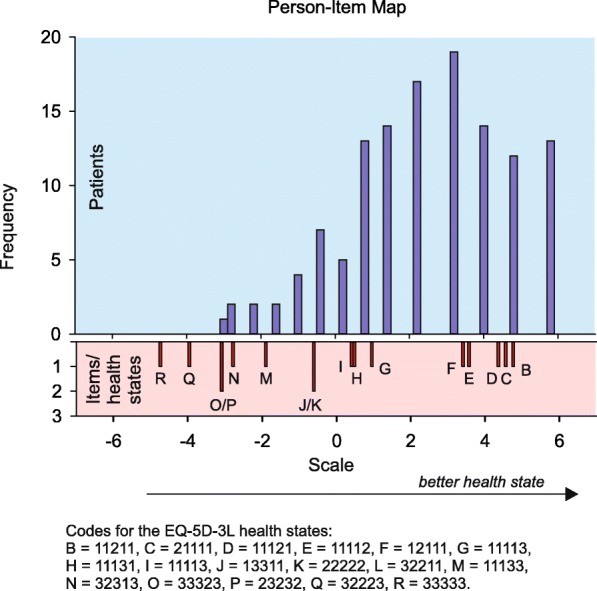
The estimated values based on the holistic MAPR model on the latent scale of the items (below: small red bars) are given next to the histogram of the person-parameter distribution (above)

The Guttman scalogram reveals that not all health states and persons are perfectly ordered (Fig. 4), this can be seen from the green dots between the red ones that indicate misfit. Given the small number of health states in relation to the small number of patients, this study showed that the Rasch (holistic MAPR) model does not hold on statistical grounds. However, after deleting health states in the analysis that were rather severe and therefore overly judged as worse than the own health conditions of the patients (states: 32211, 33323, 32223, 11133, 32313, 22222, 33333, and 23232) the holistic model does hold. An Andersen LR-test showed a log likelihood value of 7.21 with 8 dfs (*p* = 0.514). The order of the health states based on their sum score is similar to the order based on the estimates of the Rasch model. This result is as expected since the sum score is a sufficient statistic for the Rasch model. The Person-Item Map shows the distribution of patients’ own health status (the above histogram) compared to the assessed health states, see the histogram below (Fig. 5). This figure shows that more than half of the judged comparator health states were assessed as worse than the patient’s health status.

The estimated regression coefficients for MAPR model (8) reveal logical differences at all levels (Table 3). Some problems with self-care have the highest impact, followed by some problems with mood, pain, mobility, and usual activities. Severe problems with mood and pain have more impact than the other attributes. Estimates of the health states under the MAPR model (8) give almost the same order as for the Rasch model (Table 4). For the MAPR model, the pairs (11211, 21111), (11131, 11113), and (23232, 32223) have a different order and the estimated value of health state 33323 is much smaller than in the Rasch model.

**Table 3:** Parameter estimates (se) of MAPR model (Eq. 8) for the levels 2 and 3 of the five health attributes of the EQ-5D-3 L instrument

EQ-5D-3 L attributes and levels	Estimates
	α (se)
Mobility	
No problems (1)	–
Some problems (2)	−0.274 (0.229)
Confined to bed (3)	−2.909 (0.419)
Self-care	
No problems (1)	–
Some problems (2)	−1.626 (0.221)
Unable to (3)	−3.554 (0.356)
Usual activities	
No problems (1)	–
Some problems (2)	−0.548 (0.221)
Unable to (3)	−1.479 (0.307)
Pain/Discomfort	
No (1)	–
Moderate (2)	−0.752 (0.212)
Extreme (3)	−3.548 (0.282)
Depression/Anxiety	
Not (1)	–
Moderately (2)	−1.527 (0.217)
Extremely (3)	−3.352 (0.274)

**Table 4:** Comparison of sum score, health-state estimates based on Rasch (holistic MAPR) model, MAPR model (LLTM). The absolute differences in outcome between the Rasch model (holistic MAPR) and the LLTM (MAPR) model are due to scaling and should be ignored

EQ-5D-3 L health state^a^	Sum score	Rasch (Holistic MAPR) model	MAPR (LLTM) model
11111	2	^b^	0.000
11211	57	4.761	−0.548
21111	60	4.555	−0.274
11121	63	4.358	−0.752
11112	76	3.575	− 1.527
12111	79	3.406	−1.626
11113	122	0.956	−3.006
11131	129	0.482	−3.548
11113	130	0.410	−3.352
13311	142	−0.605	−4.306
22222	142	−0.605	−4.728
32211	151	−1.733	−5.083
11133	152	−1.903	−6.900
32313	156	−2.780	−9.366
33323	157	−3.087	−12.045
23232	157	−3.087	−9.451
32223	159	−3.960	−9.187
33333	160	−4.742	−14.841

^a^Code is representing the five attributes, each with three levels, of the EQ-5D-3 L instrument

^b^No estimate is obtained since the data matrix is ill-conditioned when this state is included

When comparing the conditional likelihood for the Rasch model and the MAPR model, we found a statistical difference (LR statistic = 87.9; df = 6; *p* < 0.001). This means that the goodness of fit of the MAPR model is lower than for the Rasch (the holistic MAPR) model. However, the correlation between the item parameters as estimated with the Rasch model and the item parameters of MAPR model is 0.93, so even the elaborated MAPR model performs well in explaining the item parameters. In 88.2% of the comparisons, the observed preferences agree with the predicted preferences based on the MAPR model. The kappa coefficient equals 0.71 (CI: 0.68–0.74), which is considered fair to good.

## Discussion

This article presents a novel approach to measuring health: the multi-attribute preference response model (MAPR). It was developed to quantify health states and patients’ own health status on the same unidimensional scale. The response mechanism of this model is insensitive to various biases (e.g., time preference, risk aversion, indifference procedure) that arise with conventional methods (i.e., standard gamble, time trade-off) to derive values for health states. Moreover, this is the first generic health preference-based model that fully reflects perception and reporting by patients. Besides being grounded in measurement theory, the response tasks are attractive and easy to perform in a self-completion setting.

The small study that we conducted to illustrate the procedure and results of our proposed method to measure the quality of health states and patients’ own health status showed confirming results. Although the sample size of our empirical study was very modest for performing an item response theory analysis as done here, the estimated regression weights showed a clear logical structure. Values for the small set of health states included in this study could be computed and showed a valid order of the health states as well as interpretable distances between the health states, compared with results from previous large studies based on conventional measurement methods. Note that in this small study a fixed set of only 17 health states were used and therefore we could not include interaction terms in the regression equation. In the full operational MAPR model patients will not be confronted with a fixed set of states, but with a smaller set of health-state descriptions that are closely similar to their own health status. This will lead to more efficient and robust estimation of the parameters.

The MAPR model largely eliminates unwanted mechanisms affecting valuations of health states. Prominent among these is adaptation. Health-state values derived by conventional methods are typically higher when elicited from patients, particularly those with chronic illness or disability, than from non-patients who only imagine themselves in hypothetical health conditions. Adaptation is manifest in almost all standard methods of health measurement, particularly in multi-domain instruments, often based on Likert scales as developed within the setting of classical test theory [31]. Moreover, conventional methods for valuing health states stemming from economics (e.g., standard gamble, time trade-off) are also complex and require abstract reasoning skills. These drawbacks can now be averted. Measurement with the MAPR model is based on a discrimination principle: a patient’s own health status serves as a comparator state against other (comparator) states. This indirect approach to derive values for health states is different from the conventional valuation techniques used by health economists. These valuation techniques (e.g., standard gamble, time trade-off) request a direct and absolute score (monadic measurement). Because the response task in the MAPR model is simply a preference (rank order) between a patient’s own health status (that serves as a reference standard) and a (closely) related hypothetical health state, the assessment is less likely affected by ‘subjective’ motives and easier to accomplish. Patients don’t quantity their own health status, they only compare it and rank it. This mode of measurement largely prevents biases such as adaptation and coping. From a theoretical and a practical point of view, the MAPR is more attractive than the existing valuation methods, particularly because both the judgmental task and the analysis are executed within one unifying framework.

A downside of the MAPR model is that it produces relative positions of health states. For application in DALYs and QALYs, however, MAPR-derived values need to be rescaled around the position where states are considered to become worse than dead (position of dead = 0) [32, 33]. In conventional valuation methods, ‘dead’ is not only an element of the task itself but also introduces many methodological and practical problems. Separate exercises are needed to localize the position of ‘dead’ for the MAPR model. However, recent studies suggest promising solutions; separate studies can be conducted to localize the juncture where health states are considered worse than dead [34, 35]. Such additional studies probably are better be worked out based on the input from a sample of the general population, instead of patients.

When applying any conventional Rasch model to derive metric measures, it is assumed that the underlying phenomena can be represented on a unidimensional scale. However, this crucial assumption may be questionable when quantifying a subjective phenomenon such as health, a construct with a rather broad scope. Nevertheless, the overall assumption is that health outcomes such as health status, health-related quality of life, and well-being are unidimensional concepts. Of course, this is true of all data to some extent. As many researchers have convincingly argued, unidimensionality does not imply only one factor or dimension. Rather, it implies the presence of a dominant dimension and possibly of minor dimensions that do not affect the dominant one and the unidimensionality of the model is therefore a reflection of the assumed unidimensionality of the majority of assessments we use [36]. Our health model is comparable to widely accepted models of intelligence. Typically, cognitive abilities are represented as a three-level hierarchy with numerous narrow factors at the bottom, a handful of broad, more general factors at the intermediate level, and at the apex a single factor, the *g* factor, which stands for the variance common to all cognitive tasks [37].

The MAPR model can even be extended to offer respondents a large set of candidate attributes (far more than the traditional four to nine attributes in existing instruments). An individual patient could then select those most relevant to his or her assessment. By breaking the fixed-set mold, this MAPR variant leads to a truly patient-centered preference-based health measurement approach. An extended MAPR model would most likely require thousands and thousands of responses from patients. Some solutions for this problem have already been introduced [22, 38, 39].

An alternative method to quantify health states may be the discrete choice (DC) model [40, 41]. It is based on (paired) comparisons of two or more hypothetical health states (and not a person’s health status itself). In that sense, the difference between the MAPR model and the DC model seems only minor, but in fact it is significant. The DC model only scales health states, not respondents (patients).

Several elements related to the MAPR model must be investigated empirically to confirm the assumptions underpinning it and explore its potential limitations. In particular, the data should show a Guttman structure. As data for the MAPR model is collected in patient groups, suboptimal response data may result. This may be due to problems with interpreting the health attributes and their levels, from taking cognitive shortcuts and other factors.

## Conclusions

This new patient-reported health IRT model can be used as a coherent measurement method and has a profound connection to measurement theories. Apart from developing instruments that can be used in medical settings, the MAPR model can also be used to develop health-outcome instruments to measure in health care. Operated by dedicated data collection technology with interactive routines, data capturing based on this new measurement method becomes simple and even certain distinct patient populations can be easily approached (e.g., children, elderly). In principle, the MAPR model may be suitable to measure other unidimensional phenomena such as well-being, capabilities, and other subjective attributes that are essentially based on quality [42].

## Additional file

Additional file 1: Classification of the patients in the three studies of their own health status on the EQ-5D-3 L. (DOCX 21 kb)

## Abbreviations

CML: Conditional Maximum Likelihood
CVA: Cerebrovascular Accident
DC: Discrete Choice
HrQoL: Health related Quality of Life
IBD: Inflammatory Bowel Disease
IRT: Item Response Theory
LLTM: Linear Logistic Test Model
MAPR: Multi-Attribute Preference Response
MML: Marginal Maximum Likelihood.
OPLM: One-Parameter Logistic Model.
RA: Rheumatoid Arthritis.
WHO: World Health Organization.

## Footnotes

1 For example, suppose that two attributes take values 1 to 3. Thus *x*_*i*_ = (*x*_*i*1_, *x*_*i*2_) ∈ {1, 2, 3} × {1, 2, 3}. Since *x*_2_=(1,2) is worse than *x*_1_=(1,1), we obtain an ordering in the alphas. Due to the ordering we have *β*_1_ < *β*_2_, which implies *β*_1_ = *α*_1, 1_ + *α*_2, 1_ < *α*_1, 1_ + *α*_2, 2_ = *β*_2_ and thus ${\alpha}_{j,{k}_1}<{\alpha}_{j,{k}_2}$when *k*_1_ < *k*_2_.

## References

[1] WHO LU-WHO. Preamble to the Constitution of the World Health Organization as adopted by the International Health Conference, New York, 19-22 June, 1946. Geneva, Switzerland. http://whqlibdoc.who.int/hist/official_records/constitution.pdf.

[2] Krabbe PFM (2016). The Measurement of health and Health Status: Concepts, Methods and Applications from a Multidisciplinary Perspective.

[3] Nord E, Enge AU, Gundersen V (2010). QALYs: is the value of treatment proportional to the size of the health gain?. Health Econ.

[4] Salomon JA (2014). Techniques for valuing health states. Encyclopedia of Health Economics.

[5] Attema AE, Edelaar-Peters Y, Versteegh MM, Stolk EA (2013). Time trade-off: one methodology, different methods. Eur J Health Econ.

[6] Brazier JE, Dixon S, Ratcliffe J. The role of patient preferences in cost-effectiveness analysis: a conflict of values. PharmacoEconomics. 2009;27 10.2165/11314840-000000000-00000 LB - Brazier2009. 10.2165/11314840-000000000-00000 19757864

[7] Krabbe PFM, Tromp N, Ruers TJM, van Riel PLCM (2011). Are patients’ judgments of health status really different from the general population. Heal Qual Life Outcomes.

[8] Krabbe PFM. A generalized measurement model to quantify health: the multi-attribute preference response model. PLoS One. 2013;8 10.1371/journal.pone.0079494 PMC3836915 24278141

[9] Rasch G (1980). Probabilistic models for some intelligence and attainment tests expanded edition with foreword and afterword by B.D. Wright.

[10] Fischer GH (1974). Einführung in die Theorie Psychologischer Tests. Bern: Verlag Hans Huber.

[11] Andrich D (1985). An elaboration of Guttman scaling with Rasch models for measurement. Sociol Methodol.

[12] Dolan P (1997). Modeling valuation for EuroQol health states. Med Care.

[13] Sintonen H (2001). The 15D instrument of health-related quality of life: properties and applications. Ann Intern Med.

[14] Richardson J, Sinha K, Iezzi A, khan MANV-63 MA. Modelling the utility of health states with the assessment of quality of life (AQoL) 8D instrument: overview and utility scoring algorithm. Centre for Health Econ. 2011;

[15] Anderson JP, Kaplan RM, Berry CC, Bush JW, Rumbaut RG (1989). Interday reliability of function assessment for a health status measure. The Quality of Well-being Scale Med Care.

[16] Feeny D, Furlong W, Torrance GW, Goldsmith CH, Zhu Z, DePauw S (2002). Multiattribute and single-attribute utility functions for the health utilities index mark 3 system. Med Care.

[17] Scheiblechner H (1972). Das Lernen und Lösen komplexer Denkaufgaben. Zeitschrift für Exp und Angew Psychol.

[18] Fischer GH (1973). Linear logistic test model as an instrument in educational research. Acta Psychol.

[19] Fischer GH (1983). Logistic latent trait models with linear constraints. Psychometrika.

[20] de Boeck P, Wilson M. Explanatory item response models: a generalized linear and nonlinear approach. New York: Springer-Verlag.

[21] Scheerens J, Glas C, Thomas SM. Educational evaluation, assessment and monitoring (2003). A systematic approach. New York: Taylor & Francis.

[22] Fox JP (2010). Bayesian item response modeling: theory and applications.

[23] Parkin D, Devlin NJ, Foo Y (2016). What determines the shape of an EQ-5D index distribution. Med Decis Mak.

[24] Andersen EB (1977). Sufficient statistics and latent trait models. Psychometrika Psychometrica.

[25] Fischer GH (1981). On the existence and uniqueness of maximum-likelihood estimates in the Rasch model. Psychometrica..

[26] Fischer GH, Hambleton RK, Van der WJ LI (1997). Unidimensional linear logistic Rasch models. Handbook of modern item response theory.

[27] Fischer GH (2005). Linear logistic test models. Encyclopedia of social measurement.

[28] Andersen EB (1973). A goodness of fit test for the Rasch model. Psychometrica.

[29] Fleiss JL (1981). Statistical methods for rates and proportions.

[30] Mair P, Hatzinger R. Mair MJ. eRm: extended Rasch modeling [computer software]. R package version. 0:15–4. http://cran.r-project.org/package=eRm

[31] Streiner DL, Norman GR, Cairney J (2015). Health measurement scales: a practical guide to their development and use.

[32] Weinstein M, Torrance G, McGuire A. QALYs: the basics. Value Heal 2009;12 Supplement:55–59. 10.1111/j.1524-4733.2009.00515.x 19250132

[33] Fanshel S, Bush J (1970). A health-status index and its application to health-services outcomes. Oper Res.

[34] Scalone L, Stalmeier PFM, Milani S, Krabbe PFM (2015). Values for health states with different life durations. Eur J Health Econ.

[35] van Hoorn RA, Donders ART, Oppe M, Stalmeier PM (2014). The better than dead method: feasibility and interpretation of a valuation study. PharmacoEconomics.

[36] Panayides P, Robinson C, Tymms P (2015). Rasch measurement: a response to Goldstein. Br Educ Res J.

[37] Neisser UBG, Boodoo GBTJ, Bouchard TJ, Boykin AW, Brody N, Ceci SJ (1996). Intelligence: knowns and unknowns. Am Psychol.

[38] Albert JH (1992). Bayesian estimation of normal ogive item response curves using Gibbs sampling. J Educ Stat.

[39] Chrzan K (2010). Using partial profile choice experiments to handle large numbers of attributes. Int J Mark Res.

[40] Bansback N, Brazier JE, Tsuchiya A, Anis A (2012). Using a discrete choice experiment to estimate health state utility values. J Health Econ.

[41] Krabbe PFM, Devlin NJ, Stolk EA, Shah KK, Oppe M, van Hout B (2014). Multinational evidence on the feasibility and consistency of a discrete choice model in quantifying EQ-5D-5L health states. Med Care.

[42] Barofsky I (2012). Quality: its definition and measurement as applied to the medically ill.

