# Supplementary material for: A preference-based item response theory model to measure health: concept and mathematics of the multi-attribute preference response model
Source: BMC Med Res Methodol. 2018 Jun 22;18:62. doi: 10.1186/s12874-018-0516-8 (PMC6013962; doi:10.1186/s12874-018-0516-8)
Supplement: Supplementary file 1 — Classification of the patients in the three studies of their own health status on the EQ-5D-3 L. (DOCX 21 kb) [file 12874_2018_516_MOESM1_ESM.docx]

Additional file 1

**Table:**Classification of the patients in the three studies of their own health status on the EQ-5D-3L.

| EQ-5D-3L classification | Radboud  (n=75) | MST  (n=35) | UMCG  (n=53) |
| --- | --- | --- | --- |
| 11111 | 21 | 6 | 9 |
| 11112 | 4 | 1 |  |
| 11121 | 6 | 2 | 7 |
| 11122 |  | 1 | 1 |
| 11211 | 2 | 2 | 1 |
| 11212 | 1 |  | 1 |
| 11213 |  |  | 1 |
| 11221 | 5 | 3 | 3 |
| 11222 | 5 | 4 | 4 |
| 11311 |  |  | 1 |
| 12111 |  |  | 1 |
| 12212 |  | 1 |  |
| 12221 | 1 |  |  |
| 21111 | 5 |  |  |
| 21121 | 1 | 1 | 5 |
| 21122 | 1 |  | 1 |
| 21211 |  | 3 |  |
| 21221 | 10 | 6 | 1 |
| 21222 | 2 | 1 | 2 |
| 21231 |  | 1 |  |
| 21233 |  |  | 1 |
| 21321 |  |  | 1 |
| 21323 |  |  | 1 |
| 22111 |  |  | 1 |
| 22122 |  | 1 |  |
| 22211 | 4 |  | 1 |
| 22223 |  |  | 1 |
| 22231 |  |  | 1 |
| 22232 | 2 |  | 1 |
| 22233 |  |  | 1 |
| 22311 | 1 |  |  |
| 22321 | 1 | 1 |  |
| 22322 |  | 1 |  |
| 22331 | 1 |  |  |
| 22332 | 1 |  |  |
| 32111 |  |  | 1 |
| 32221 |  |  | 1 |
| 32222 |  |  | 1 |
| 33211 |  |  | 1 |
| 33221 |  |  | 1 |
| 33222 |  |  | 1 |
